# Advanced Intra-Thoracic Ultrasound-Guided Thoracoscopic Lung Resection: A Case Report

**DOI:** 10.70352/scrj.cr.25-0269

**Published:** 2025-09-19

**Authors:** Yukiko Matsui, Takahide Toyoda, Yuki Sata, Terunaga Inage, Kazuhisa Tanaka, Hajime Tamura, Masako Chiyo, Kaito Nakama, Takashi Kishimoto, Jun-ichiro Ikeda, Hidemi Suzuki

**Affiliations:** 1 Department of General Thoracic Surgery, Chiba University Graduate School of Medicine, Chiba, Chiba, Japan; 2 Department of Diagnostic Pathology, Chiba University Graduate School of Medicine, Chiba, Chiba, Japan; 3 Department of Molecular Pathology, Chiba University Graduate School of Medicine, Chiba, Chiba, Japan

**Keywords:** intra-thoracic ultrasound, minimally invasive surgery, lesion identification

## Abstract

**INTRODUCTION:**

Minimally invasive surgery has recently become the standard approach for pulmonary lesion resection, and its usefulness has been reported. However, these techniques involve small incisions, making intraoperative palpation difficult. Therefore, a simple and minimally invasive method is required to identify these lesions. Herein, we present a case of thoracoscopic lung wedge resection in which intra-thoracic ultrasonography equipped with a new technology was successfully used for lesion localization.

**CASE PRESENTATION:**

A male patient in his 60s presented with a right intrapulmonary nodule following chemoradiotherapy for oropharyngeal cancer. Surgical resection was planned for the suspected malignancy. The procedure was a single-port thoracoscopic wedge resection of the right upper lobe. Intraoperatively, intra-thoracic ultrasonography successfully identified a pulmonary lesion, eliminating the need for palpation. Histopathological examination confirmed squamous cell carcinoma and complete resection was achieved.

**CONCLUSIONS:**

We successfully performed a single-port thoracoscopic pulmonary wedge resection using intraoperative thoracic ultrasonography for precise lesion localization. The ultrasound devices we used were equipped with advanced technology, enabling clearer images. This case highlights the potential utility of intra-thoracic ultrasonography in minimally invasive lung surgery, as it enables accurate lesion localization and successful resection.

## Abbreviations

AI
: artificial intelligence
CBCT
: cone-beam computed tomography
RATS
: robot-assisted thoracoscopic surgery
VATS
: video-assisted thoracoscopic surgery

## INTRODUCTION

Minimally invasive surgeries, such as video-assisted thoracoscopic surgery (VATS) and robot-assisted thoracoscopic surgery (RATS), have become standard approaches for pulmonary lesion resection, and their usefulness has been reported.^1)^ However, these techniques require small incisions, making intraoperative palpation challenging. To compensate for the inability to palpate lesions, alternative intraoperative localization methods, such as cone-beam computed tomography (CBCT) and radiofrequency identification, have been explored.^2,3)^ These methods require specialized equipment available only at select institutions, and CBCT involves radiation exposure, which is an additional concern. Therefore, a simpler and less invasive method for intraoperative lesion identification is desirable.

Since its introduction to medical diagnostics in the 1950s,^4)^ ultrasound technology has undergone remarkable advancements and has become an indispensable tool in surgical practice. Recently, intraoperative ultrasonography has been increasingly used for diagnostic purposes.^5)^ Although several reports have described intra-thoracic ultrasound-assisted lung resection,^6–8)^ its widespread adoption remains limited. Here, we present a case of VATS wedge resection in which intra-thoracic ultrasonography is enhanced with advanced technology. The device we used was the ARIETTA 850 DeepInsight (FUJIFILM, Tokyo, Japan), designed using artificial intelligence (AI) technology to accurately distinguish weak ultrasound signals from electrical noise and generate stable images. This AI technology can effectively distinguish between weak ultrasound signals such as speckle signals and electrical noise, leading to highly accurate recognition and clear imaging. We also used the laparoscopic ultrasound probe L43K (FUJIFILM), a drop-in probe and expected to improve spatial resolution due to its high frequency, which was introduced through the port and directly applied to the lung surface.

## CASE PRESENTATION

**Fig. 1:**
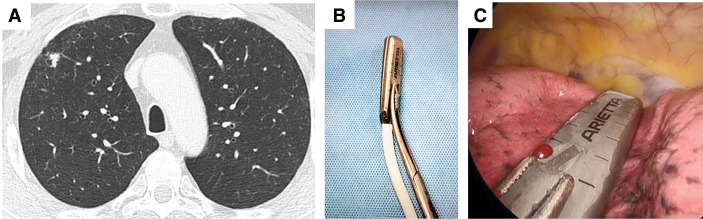
Preoperative and intraoperative findings. (**A**) Preoperative CT image showing a 1.1-cm nodule in the apical segment (S1) of the right lung, located 0.9 cm away from the pleural surface. (**B**) The back of the probe was grasped with forceps. (**C**) Intraoperative ultrasound with a probe placed directly on the lung surface.

A male patient in his 60s underwent chemoradiotherapy for oropharyngeal cancer 2 years prior to presentation. During follow-up, CT revealed a 1.1 cm nodule in the apical segment (S1) of the right lung. It was located 0.9 cm away from the pleural surface (**Fig. 1A**). Surgical resection was planned for the suspected primary lung malignancy. Before surgery, the use of a laparoscopic ultrasound device in the thoracic cavity was reviewed and approved by the Institutional Review Board of our institution (Approval #: 03-31). The patient was informed of the procedure and provided written informed consent.

Surgery was performed under general anesthesia with single-lung ventilation. A single-port VATS wedge resection of the right upper lobe was performed. A 3-cm incision was made along the midaxillary line in the right 6th intercostal space. Following lung deflation, intraoperative ultrasound examination was performed using the devices mentioned above (**Fig. 1B**, **1C**).

**Fig. 2:**
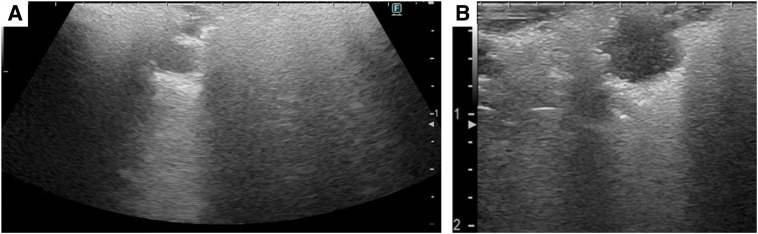
Intraoperative ultrasound imaging. (**A**) The pulmonary lesion appeared hypoechoic on intraoperative ultrasonography, with the deepest portion exhibiting hyperechoic characteristics. (**B**) Ultrasound examination of the resected lung confirmed the tumor.

**Fig. 3:**
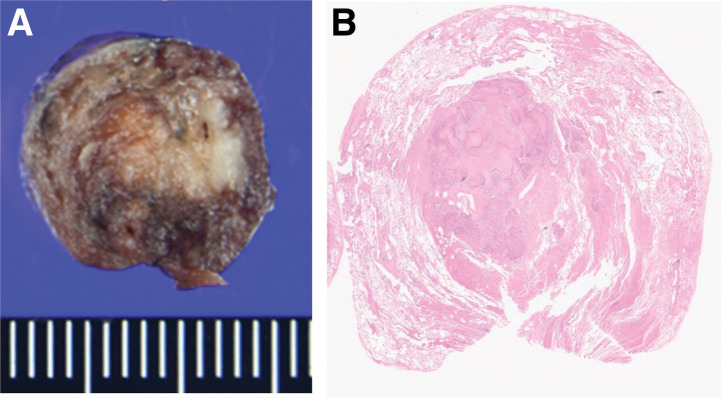
Histopathological findings. (**A**) Gross appearance of the resected specimen. (**B**) Low-power view of the resected specimen.

The pulmonary lesion was identified using ultrasound imaging, and it generally appeared hypoechoic, with the deepest part of the lesion showing hyperechoic findings with hyperechoic shadow (**Fig. 2A**). The hyperechoic shadow is a common finding on the dorsal surface of the tumor. It is due to the difference in transmission and reflection of sound waves between the tumor lesion and the surrounding lung. We used forceps to grasp the tip of the probe during thoracoscopic manipulation. The tumor location and depth were clearly visualized, enabling precise lesion identification without palpation. No pleural surface marking was performed. Wedge resection of the lung was performed based solely on ultrasound findings. After localization and depth by ultrasound, resection was performed grasping the lung surrounding the lesion with forceps to ensure an adequate margin. The resection margin distance was confirmed by visual inspection. The resection margin was 1 cm. Ultrasonography was also performed on the resected specimen in the operative field immediately and confirmed that the tumor was completely removed (**Fig. 2B**). Because of the possibility of metastatic lung tumor, a wedge resection was performed. A 19 Fr drain tube was placed before the end of the surgery. The postoperative course was uneventful, and the patient was discharged on POD 7. The tumor diameter was 1.1 cm. Histopathological examination confirmed the diagnosis of squamous cell carcinoma. The resection margin, after staple removal, was about 10 mm with no malignant findings at the surgical margins, indicating complete resection (**Fig. 3A** and **3B**). As for postoperative management since the final pathology result was primary lung cancer, we evaluated the need for additional resection. After explaining the contents of the consideration to the patient, he requested follow-up observation.

## DISCUSSION

Medical ultrasound devices are widely used in clinical practice because of their convenience and versatility. Ultrasonography is commonly used for lesion identification and treatment during intra-abdominal surgery, particularly for hepatic and renal lesions.^4)^ Compact ultrasound probes are extensively used in laparoscopic surgery to facilitate intra-operative imaging.^5)^ However, in thoracoscopic surgery for pulmonary lesions, air within the lungs has traditionally been considered a major limitation, making ultrasound imaging of intrapulmonary lesions difficult. Although there have been reports of lung resection using an intra-thoracic ultrasound device,^6–8)^ it has not become widespread. This may be due to the low resolution caused by air in the lungs. Therefore, we attempted to use a device equipped with this new technology. In this case, we performed the surgery under one-lung ventilation and further compressed the lung with forceps to collapse the right lung as much as possible to perform the ultrasound examination with less air. In addition, an image enhancement algorithm based on deep learning further improved the visualization of the nodule.

Palpation plays an important role in identifying pulmonary lesions during pulmonary surgery,^9)^ especially in cases in which nodular lesions are not visible on the visceral pleural surface. Direct tactile feedback is essential for confirming the location of a lesion. However, palpation can be difficult in minimally invasive procedures, such as VATS and RATS, in which surgery is performed through small incisions. Therefore, an extension of the incision may be necessary if palpation is required. Compared with traditional open thoracotomy, VATS has been associated with improved QOL, reduced postoperative complications, shorter hospital stays, and decreased postoperative pain.^10,11)^ However, extending the incision to allow palpation may increase surgical invasiveness, potentially negating the benefits of minimally invasive surgery.

Ultrasound systems have the advantage of being widely available. Currently, methods of lung nodule identification other than palpation in lung surgery, such as virtual-assisted lung mapping, cone-beam CT, and high-frequency identification, are in practical use, and all of these methods are excellent.^2,3,12)^ The effectiveness of these methods is considered equivalent to this method. However, they have limitations, such as the need for high-performance equipment, which can only be used in limited facilities, and the use of CT, which increases radiation exposure. On the other hand, ultrasound devices are widely used and have the advantage of no radiation exposure. In addition, although ultrasound devices are expensive, they are cost-effective because they are applied to the lungs from devices used in other organ surgeries, such as liver and kidney surgery. The potential disadvantages of this method include that the lesion detection rate depends on the condition of the lesion, the intensity of the emphysema, and the skills of the surgeons.

The use of intra-thoracic ultrasound during pulmonary resection has been previously reported.^7,8)^ In this patient, we successfully identified intrapulmonary nodular lesions intraoperatively using ultrasonography equipped with a new technology while the lungs were deflated. Consequently, we accurately identified the lesion location without relying on palpation. This is more important when using a less invasive procedure, such as single-port VATS. In the future, we plan to include more cases to evaluate the utility of intra-operative thoracic ultrasonography for pulmonary lesion identification and its potential role in thoracoscopic surgery.

## CONCLUSIONS

We successfully performed a single-port thoracoscopic pulmonary wedge resection using advanced intraoperative thoracic ultrasonography equipped with a new technology for precise lesion localization. The ultrasound devices we used were equipped with advanced technology, enabling clearer images. This case highlights the potential utility of intra-operative thoracic ultrasonography in minimally invasive lung surgery and provides a simple and effective alternative for pulmonary lesion identification.

## DECLARATIONS

### Availability of data and materials

The datasets supporting the conclusions of this article are included within the article and its additional files.
